# Use of N-hexyl Cyanoacrylate Monomers for the Treatment of Intra- and Extracranial Arteriovenous Malformations: A Single-Center Experience

**DOI:** 10.7759/cureus.84290

**Published:** 2025-05-17

**Authors:** Eduardo Murias Quintana, José Rodríguez Castro, Josep Puig, Alberto Gil García, René Chapot, Víctor Maestro, Juan Carlos Llibre, Julio Cesar Gutiérrez Morales, Faustino García Arias, Pedro Vega

**Affiliations:** 1 Interventional Neuroradiology, Hospital Universitario Central de Asturias, Oviedo, ESP; 2 Interventional Neuroradiology Chair of the University of Oviedo (CENIT), Universidad de Oviedo, Oviedo, ESP; 3 Interventional Neuroradiology, Hospital Clínic de Barcelona, Barcelona, ESP; 4 Interventional Neuroradiology, Hospital Universitario Marqués de Valdecilla, Santander, ESP; 5 Interventional Neuroradiology, Alfried Krupp Krankenhaus, Essen, DEU; 6 Interventional Neuroradiology, Hospital Universitario de Salamanca, Salamanca, ESP; 7 Neurosurgery, Hospital Universitario Central de Asturias, Oviedo, ESP; 8 Musculoskeletal Imaging, Hospital Universitario Central de Asturias, Oviedo, ESP

**Keywords:** embolic tool, endovascular treatment, nhca (n-hexyl cyanoacrylate) monomers, safety, vascular malformations

## Abstract

Purpose: Liquid embolic agents (LEAs) are commonly used in the endovascular treatment of vascular malformations for their penetration, visibility, and reflux control. This study presents our experience with N-hexyl cyanoacrylate (NHCA), also known as "Magic Glue," and its strengths and limitations. Methods: A retrospective single-center case series study was conducted to assess outcomes among patients with complex head and neck vascular lesions treated with NHCA injection at Hospital Universitario Central de Asturias, Oviedo, Spain, between January 2022 and December 2023. Procedure and outcome measures included justification for using NHCA, technical success (defined as the outcome achieved based on objective), materials used for catheterization, dilution ratio of NHCA, and observations of technical significance during the procedure.

Results: A total of 24 NHCA injections were performed in 22 patients with intra- and extracranial vascular lesions. An arteriovenous malformation (AVM) was treated in 83.3% (20/24) of cases, including nidal pseudoaneurysms in ruptured AVMs (8/24, 33.3%), arterial feeder embolization in AVM treatment (7/24, 29.2%), and single-feeder AVMs (5/24, 20.8%). The remaining cases included bone or skin vascular malformations (2/24, 8.3%) and dural arteriovenous fistulas (2/24, 8.3%). In 87.5% (21/24) of cases, procedures were successful using a 1:4 dilution ratio of NHCA in lipiodol. In 70.8% (17/24) of cases, the HeadWay Duo 167 cm microcatheter was used to gain arterial access, and the Magic 1.2F 165 cm microcatheter was used in the remaining cases. Injection durations ranged from 27 seconds to 9 minutes and 38 seconds. No clinically significant complications occurred, and microcatheter retrieval was successful in 100% of cases. Complete occlusion with a single NHCA injection into the arterial feeder was achieved in 12.5% (3/24) of cases. NHCA demonstrated optimal penetration and advancement capacity in both intranidal and proximal injections, allowing effective embolization of complex vascular structures. Conclusion: NHCA demonstrated safe and effective penetration and advancement in both intranidal and proximal injections, allowing successful embolization of complex vascular structures. Further research is necessary to establish its long-term efficacy and optimize its clinical applications.

## Introduction

Ethylene vinyl alcohols (EVOHs) and cyanoacrylates (CYAs) are two types of liquid embolic agents (LEAs) commonly used to treat vascular malformations of the head and neck. CYAs were the first agents introduced for endovascular embolization. However, due to their rapid polymerization and adhesive properties, they necessitate shorter injection times and faster microcatheter withdrawal. EVOHs with non-adhesive properties have recently emerged as an alternative, enabling prolonged injections and achieving more complete occlusions, particularly when reflux control techniques are applied [1,2].

N-hexyl cyanoacrylate (NHCA) monomers have been introduced as an alternative to N-butyl cyanoacrylate (NBCA), a commonly used CYA. Preclinical studies suggest that NHCA, commercially known as Magic Glue (manufactured by Peters Surgical, Bobigny, France and distributed by Balt Extrusion) or Purefill (Medtronic, Irvine, CA, USA), offers comparable occlusive efficacy to NBCA formulations such as Histoacryl® (B. Braun, Melsungen, Germany) and Glubran® 2 (GEM Srl, Viareggio, Italy). However, NHCA exhibits substantially lower adhesive strength, which facilitates prolonged injections and allows for safer microcatheter retrieval without the need for detachable-tip catheters [3-5]. Nevertheless, there remains a risk for delayed dislodgement of the embolic agent, although rare [1,2].

Cyanoacrylate agents (including NBCA, NHCA, and their derivatives) vary in adhesive strength and polymerization times, which can influence their suitability for specific vascular pathologies and procedural requirements [4,6]. The use of NHCA for suturing during open and laparoscopic surgeries is well-established [5,7]. Biocompatibility and safety studies support its intravascular use [4,5]. However, studies specifically evaluating its effectiveness and clinical indications in the central nervous system remain limited [1-3]. In this descriptive case series, we present and discuss our experience with Magic Glue in a variety of head and neck vascular pathologies. By observing the product's behavior under real-world conditions, we report its benefits and limitations in different clinical indications at our center, with particular attention paid to clinical complications.

## Materials and methods

Study design and population

A retrospective single-center case series study was conducted to assess outcomes among patients with complex head and neck vascular lesions treated with NHCA injection at Hospital Universitario Central de Asturias, Oviedo, Spain, between January 2022 and December 2023. Selection criteria for treatment with NHCA injections were: (i) complex vascular malformations unsuitable for other LEAs, (ii) need for prolonged injection due to high-flow conditions, and (iii) lesions with a significant distance between the catheter tip and target. These criteria were guided by operator’s experience and lesion characteristics. Due to the retrospective nature of this study, which involved a routine interventional procedure, Institutional Review Board approval was not required. 

Procedures

All procedures were conducted under general anesthesia in a dedicated biplane Angio suite (Artis Zee, Siemens, Erlangen, Germany). Prior to the procedure, patients were partially heparinized with 2500 IU and given nimodipine (4mg) diluted in a 1-liter saline flushing solution to prevent vasospasm. The employed guiding catheter was a 5F, 6F Navien or Fargo 6F.

The choice of NHCA over NBCA was informed by clinical scenarios where NHCA's adhesive strength and penetration were considered advantageous, including high-flow arteriovenous malformations (AVMs) and cases requiring distal embolization. In particular, the 'pressure cooker technique' was avoided in cases where anatomical conditions, such as absence of a suitable reflux path or high-flow shunts, made proximal occlusion technically unfeasible. The pressure cooker technique refers to a dual-balloon or coil-assisted embolization strategy that creates a closed system to prevent reflux and promote distal filling by trapping the embolic agent between two occlusive points. While effective in certain scenarios, this technique was not applicable in our series due to anatomical and technical constraints.

Instead, we employed a continuous, pulse-free injection technique without proximal balloon occlusion or coil protection. This allowed the NHCA to advance distally under low pressure, relying on its lower adhesive strength and prolonged polymerization time to achieve deep nidus penetration and safe microcatheter retrieval. This approach was consistent with our standard NHCA delivery protocol.

Compared to NBCA, NHCA’s reduced adhesiveness permits longer injections without the need for detachable-tip microcatheters or flow-arrest techniques such as the pressure cooker. Unlike EVOH-based agents (e.g., Onyx), NHCA can achieve comparable or superior penetration in selected cases while simplifying catheter requirements and procedural setup.

Like NBCAs, the preparation of NHCA required manual mixing with iodized oil and flushing the microcatheter with glucose solution. A 1:4 (20%) dilution of NHCA in Lipiodol was used, consistent with previous clinical studies describing its application for neuroendovascular embolization [5]. The injection technique, including the position of the microcatheter and pre-formation of micros, remained consistent across all cases, except for variations in dilution. The "pressure cooker technique" was not used [7]. To maintain consistency with our usual injection technique for other cyanoacrylates, the injection mode remained continuous and pulse-free. Therefore, any observed behavioral differences could be attributed to the specific properties of NHCA rather than the injection method. To verify polymerization behavior, NHCA was injected into saline; copolymer formation was visually confirmed by changes in droplet appearance [2].

In this study, adequate reflux refers to controlled reflux that enhances embolization while avoiding non-target embolization or catheter entrapment. Significant reflux, in contrast, describes excessive reflux with potential risks.

Microcatheter selection was guided by the intrinsic characteristics of the lesions and the feeders to be treated. Thinner, smaller-profile, and longer microcatheters were preferred to facilitate navigation through complex vascular anatomy. The HeadWay Duo 167 microcatheter (Microvention, Aliso Viejo, CA), compatible with 0.014-inch guidewires, was used to achieve initial supraselective catheterization and was subsequently exchanged for 0.007- or 0.008-inch guidewires to enhance distal access. Alternatively, the Magic 1.2 microcatheter (Balt, Montmorency, France) was selected for navigation into distal and small-caliber feeders of vascular lesions due to its smaller profile and flexible design.

Procedural and outcome measures 

The following procedural and outcome measures were analyzed in this study: (i) justification for using NHCA instead of other LEAs; (ii) dilution of NHCA; (iii) materials used for catheterization; (iv) technical success/outcome achieved based on objective; and (v) observations of technical significance during the procedures.

Statistical analysis

Descriptive statistics were used to evaluate the procedural characteristics and study outcomes.

## Results

A total of 24 NHCA injections were administered to 22 patients across six clinical indications with diverse pathologies and clinical goals (Table 1). In 83.3% (20/24) of cases, the treated lesions were AVMs, including nidal pseudoaneurysms in ruptured AVMs (8/24, 33.3%), arterial feeder embolization in AVM treatment (7/24, 29.2%), and single-feeder AVMs (5/24, 20.8%). The remaining cases included bone or skin vascular malformations (2/24, 8.3%) and dural arteriovenous fistulas (2/24, 8.3%).

**Table 1:** Indications, results, and justifications for each NHCA application. † Refers to cases using a 1:4 dilution rate of NHCA in lipiodol. One procedure was performed using a 1:6 dilution and is excluded from the “Success” total. AVM: Arteriovenous malformation

Indication	Justification	Number of injections	Objective	Microcatheter	Dilution	Time of injection > 3 min	Success	Noteworthy findings
Treatment of nidal pseudoaneurysms in ruptured AVMs	Improves advance into the pseudoaneurysm	8	Pseudoaneurysm occlusion	6 HeadWay Duo 167 2 Magic 1.2 F	1:4	1/8	8/8	Excessive progression in one case, without clinical repercussions. Venous passage later after injection.
Arterial feeder embolization in AVM treatment	Occlusion of an arterial feeder far away from the nidus	7	Occlusion of the pedicle and part of the nidus	4 HeadWay Duo 167 4 HeadWay Duo 156 2 Magic 1.2 F	1:4	1/7	6/7	No liquid embolic progression in one of the cases.
Single-feeder AVM embolization	Occlusion of the nidus and main vein in micro-AVM with an arterial approach not suitable for “pressure cooker”	3	Complete occlusion of the AVM	1 HeadWay Duo 167 2 Magic 1.2 F	1:4	0/3	3/3	
Single feeder AVM embolization	Short distance for secure reflux	2	Complete occlusion of the AVM	1 HeadWay Duo 167	1:4	1/2	2/2	Venous passage later after infusion in one injection
Bone or skin vascular malformation	Impossibility of a catheterization close to the nidus	2	Complete occlusion of the preoperative AVM	1 HeadWay Duo 167 1 Magic 1.2 F	1:4 / 1:6	1/2	1/2	Advance into the bony vascular malformation
Occlusion of meningeal supply in extensive AVF of the superior sagittal sinus	Occlusion of a small calibermeningeal component far away from the dural fistula	2	Occlusion of the meningeal component of the fistula	2 HeadWay Duo 167	1:4	0/2	1/2	No distal penetration into the fistula, proximal injection
Total	-	24	-	17/24 HeadWay Duo 7/24 Magic 1.2 F	23/24 1:4 1/24 1:6	4/24	21/24^†^	No complications with neurological repercussion

Of the 24 procedures, 20 (83.3%) were performed for intracranial lesions and four (16.7%) for extracranial lesions. Technical success was observed across both groups; however, NHCA demonstrated particular benefit in intracranial procedures requiring deep nidus penetration or prolonged injections, such as posterior fossa AVMs and dural AVFs.

Injection durations ranged from 27 seconds to 9 minutes and 38 seconds, with a median of 2 minutes and 45 seconds. Four cases (16.7%) required injections exceeding three minutes, highlighting NHCA’s capacity for prolonged and controlled delivery. No clinically significant post-procedural complications or neurological deficits were observed.

NHCA was chosen for injections in vascular territories where the pressure cooker technique was inapplicable (3/24, 12.5%), when adequate reflux could not be achieved (2/24, 8.3%), or when significant advancement was desired but deemed unattainable with other materials based on the operator's experience. In 54.2% (13/24) of cases, NHCA was chosen to achieve a lengthier injection time to maximize penetration. NHCA was used in 37.5% (9/24) of cases due to challenging navigation of the microcatheter and the long distance between the catheter tip and the target embolization site. In 8.3% (2/24) of cases, NHCA was selected when adequate reflux could not be achieved, to avoid excessive reflux and associated complications. 

In 87.5% (21/24) of cases, the procedure was successful. A 1:4 dilution of NHCA in lipiodol was used in 95.8% (23/24) of procedures; a 1:6 dilution was used in one case. The duration of NHCA injection ranged from 27 seconds to 9 minutes and 38 seconds. A total of 16.7% (4/24) of procedures involved injections lasting longer than three minutes, in which NHCA was used to enhance distal penetration of the embolic agent. 

The HeadWay Duo 167 cm microcatheter was used in 70.8% (17/24) of cases to gain arterial access, and the remaining seven cases utilized the Magic 1.2F 165 cm microcatheter. The microcatheter was successfully removed in all cases without any associated hemorrhage. 

Complete occlusion of the AVM with a single NHCA injection into the arterial feeder was achieved in 12.5% (3/24) of cases. There were no clinically significant complications. 

Illustrative cases

**Figure 1:**
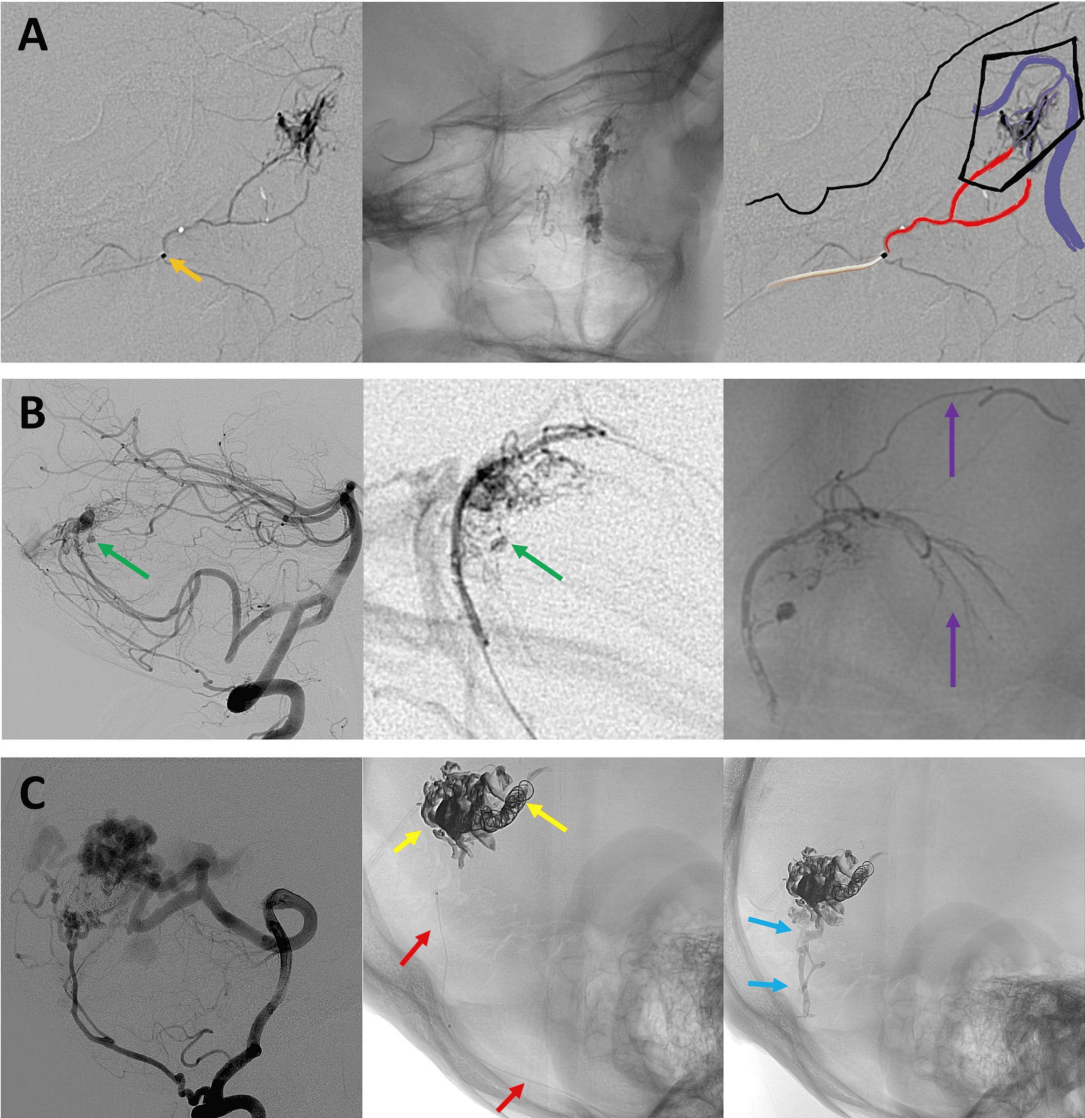
Cases 1-3 (A) Embolization of a vascular malformation in the maxillary bone. NHCA (Magic Glue) is injected into the arterial supply using a microcatheter for a prolonged duration, resulting in adequate occlusion of the malformation. (B) Partial embolization of a ruptured posterior fossa arteriovenous malformation with NHCA. Significant reflux is observed without complications during microcatheter withdrawal. The material advances into the collateral anastomotic network without clinical significance. (C) Treatment of a posterior fossa arteriovenous malformation using the pressure cooker technique. Injecting NHCA through a microcatheter allows a protracted injection time and significant reflux while permitting the safe removal of the microcatheter. NHCA: N-hexyl cyanoacrylate

**Figure 2:**
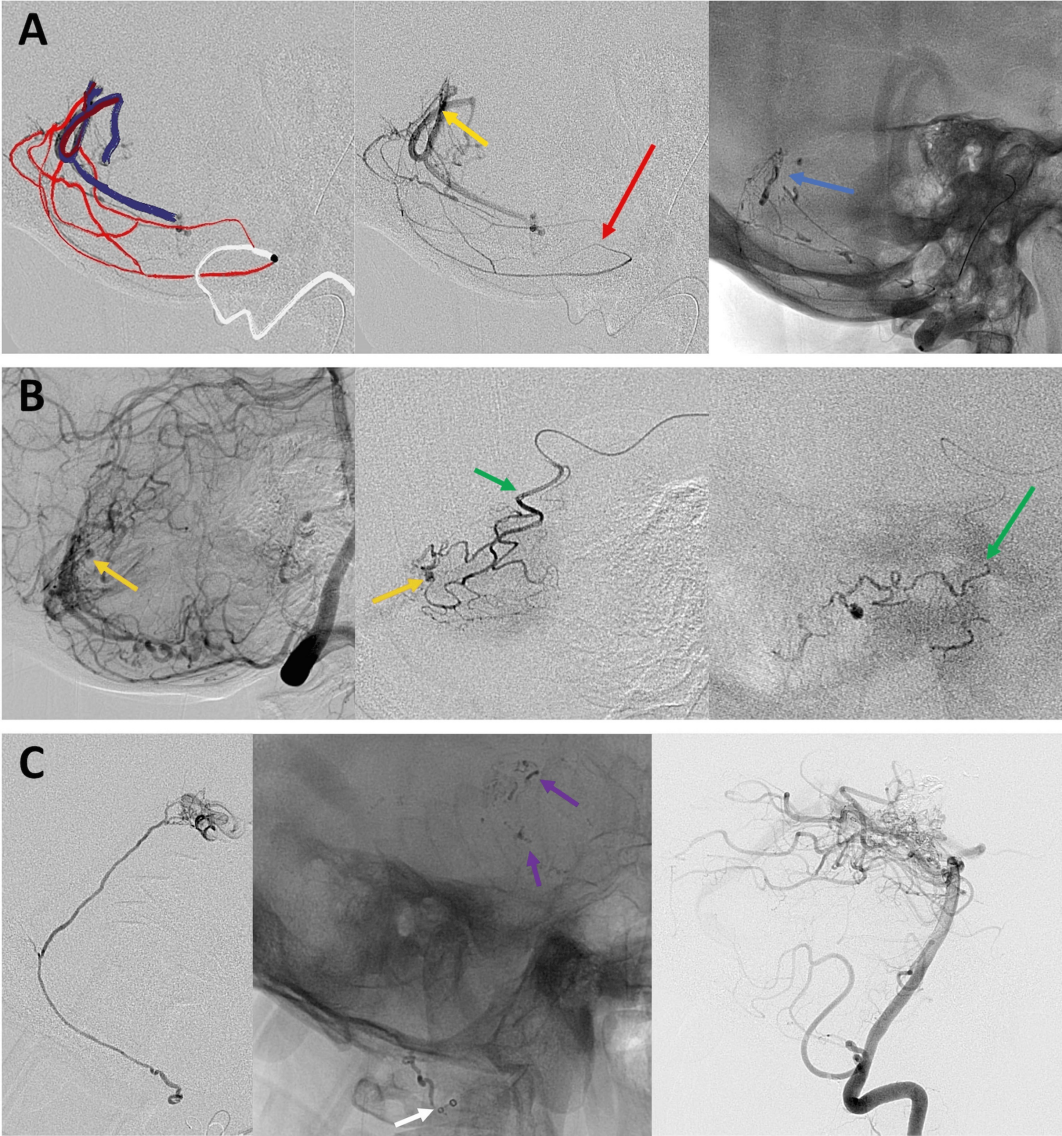
Cases 4-6 (A) Embolization of a type III arteriovenous fistula with intraparenchymal hemorrhage. NHCA is injected from a proximal position, achieving complete fistula occlusion despite inability to catheterize tiny feeders. (B) Embolization of a posterior fossa arteriovenous malformation with NHCA. The microcatheter is positioned distal to the target aneurysm, and the injection results in exclusion of the aneurysm without complications. (C) Treatment of a posterior fossa arteriovenous malformation using NHCA injection. The material advances distally, reaching the nidus of the malformation, demonstrating its penetration capacity and limited reflux. NHCA: N-hexyl cyanoacrylate

**Figure 3:**
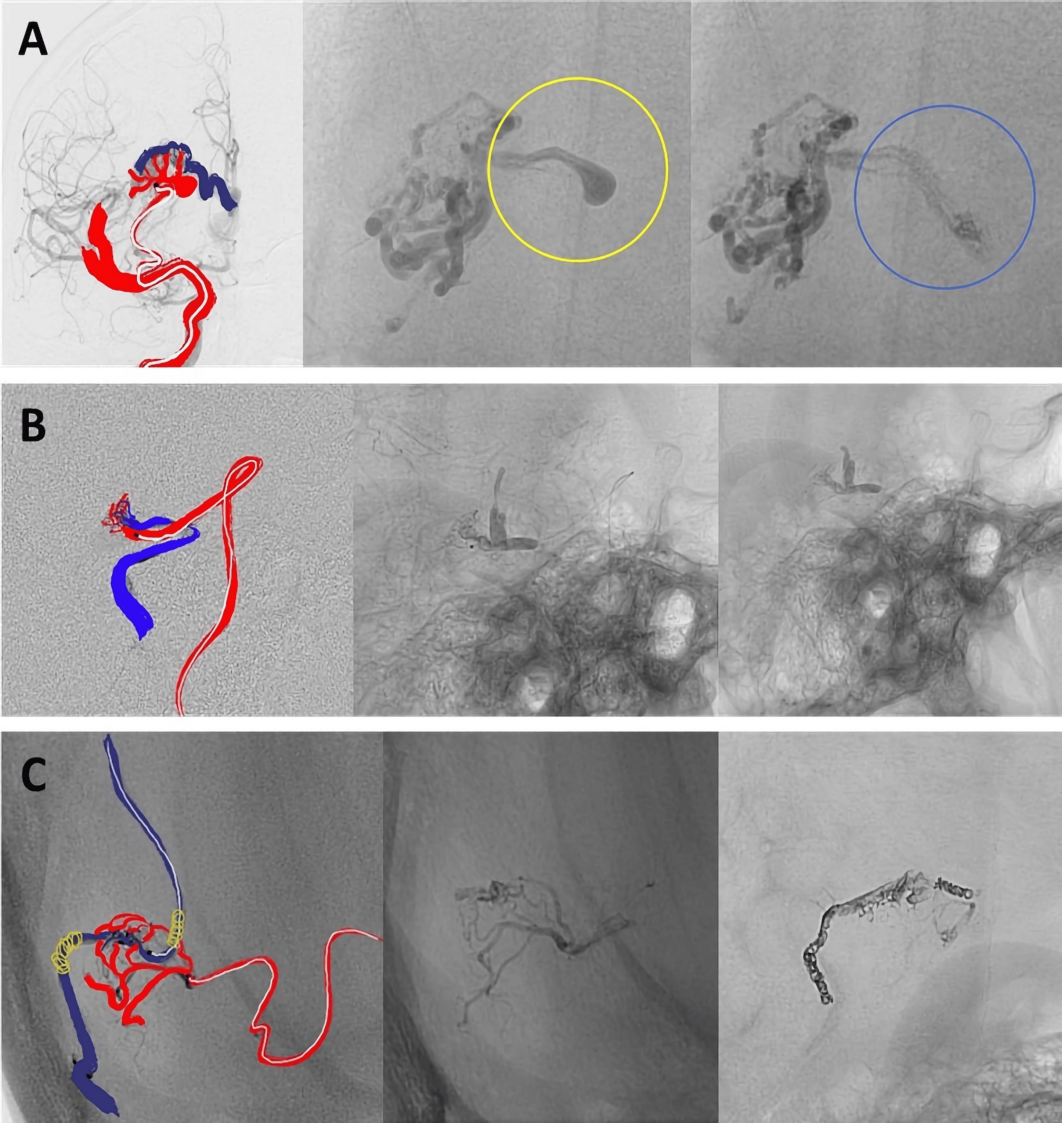
Cases 7-9 (A) Embolization of an arteriovenous malformation with a nidal pseudoaneurysm. The injection of NHCA causes the nidus and pseudoaneurysm to fill. NHCA undergoes morphological changes and fragmentation over time. (B) Incomplete treatment of an arteriovenous malformation using a venous route. NHCA is used in a subsequent procedure to close remaining portions. Long-term stability of the closure needs further evaluation. (C) Posterior fossa arteriovenous malformation with material fragmentation and displacement in the foot of the vein months after injection. NHCA: N-hexyl cyanoacrylate

Figures 1-3 present representative examples of the technical applications and outcomes observed with NHCA embolization. 

Figure 1, row A illustrates a case in which a high-flow vascular malformation of the maxillary bone was embolized through an internal maxillary branch with an injection time of 9 minutes and 38 seconds, resulting in adequate occlusion of the malformation. Figure 1, row B depicts a case in which a posterior fossa AVM with intraparenchymal hematoma and a nidal pseudoaneurysm was successfully embolized with significant reflux and an injection time exceeding three minutes, without complications. In Figure 1, row C, NHCA was used in the treatment of posterior fossa AVMs (as in Figure 1, row B). In both cases, prolonged injections and significant reflux were recorded, with successful microcatheter retrieval and no immediate clinical complications. Figure 1 shows the successful removal of a microcatheter from a lengthy meningeal branch of the posterior fossa.

In Figure 2, row A, embolization of a type III arteriovenous fistula was achieved from a proximal location despite the inability to access small-caliber feeders. In Figure 2, row B, the catheter was positioned distal to the target aneurysm, with aneurysm occlusion achieved with NHCA. Figure 2, rows A-B demonstrate NHCA injections performed at a significant distance from the treatment target, illustrating effective penetration and diffusion, with the agent even reaching the venous component of the lesions. In Figure 2, row C, distal advancement of NHCA into the nidus was observed, with limited reflux. 

Figure 3, row A shows NHCA embolization of a nidal pseudoaneurysm, followed by post-injection morphological changes and fragmentation. In Figure 3, row B, NHCA was used in a second-stage procedure following incomplete embolization via a venous route. Figure 3, row C demonstrates delayed displacement of embolic material into the venous outflow tract, identified during follow-up. 

## Discussion

In this study, the decision to use NHCA was based on its ability to address procedural challenges such as prolonged injection requirements and distal embolization targets. NHCA enabled prolonged injections, effective distal penetration, and reliable catheter retrieval across diverse vascular lesions, with no clinically significant complications. The results of this study, the largest reported clinical experience to date involving NHCA for head and neck vascular malformations, suggest that NHCA can be used safely in technically demanding procedures that require extended delivery or deeper penetration of the embolic agent. 

While NHCA proved effective in both intra- and extracranial vascular lesions, its utility was particularly notable in intracranial cases involving high-flow or complex angioarchitecture. The ability to deliver prolonged, low-pressure injections enabled deeper embolic penetration in lesions that would have been challenging to treat with other LEAs. This was especially evident in AVMs of the posterior fossa and dural AVFs with distal feeders.

While few publications have assessed NHCA in the treatment of vascular pathologies, studies in animal models and laboratory settings have investigated its use [1-5,8-11]. A preclinical swine model demonstrated that Purefill exhibited similar occlusive efficacy and distal distribution to NBCA, with lower adhesive strength and comparable histopathological findings at 90 days [3]. A clinical study reported successful embolization of four paragangliomas and one dural AVF using Magic Glue as the primary or adjunctive embolic agent [5], achieving subtotal or total devascularization in most cases without catheter entrapment. NHCA allowed slow, controlled injections with behavior similar to Onyx [5].

NHCA is distinguished from other cyanoacrylates, including NBCA, primarily by its weaker adhesive strength, a characteristic that can offer procedural advantages such as more controlled polymerization, compatibility with lower-profile catheters for accessing small or tortuous vessels, and potentially reduced toxicity due to slower degradation [4,5,12]. These properties may make NHCA preferable to other liquid embolic agents in certain clinical situations. One notable advantage is the ability to perform prolonged injections with effective advancement of the embolic agent, with injection durations exceeding one hour reported in the literature [3,4]. NHCA also enables effective embolization from distal or proximal injection sites, which is particularly useful in cases with complex or tortuous anatomy, where EVOH-based agents may be less effective due to reflux limitations or catheter positioning challenges [2].

Another distinguishing property of NHCA is its ability to penetrate and advance through both intranidal and proximal injection sites, even when targeting locations far from the treatment site. This property supports the use of NHCA, particularly in distal lesions where the embolic agent needs to navigate small angioarchitecture, such as in nidal pseudoaneurysms, or when reaching the target vein in dural arteriovenous fistulas [1]. 

Although fragmentation of the polymer was observed during subsequent injections in this study, complete closure of the target for embolization was achieved. This suggests that NHCA's polymerization behavior, which may be prone to fragmentation under certain conditions, does not compromise complete closure, and may be advantageous in treating complex or high-flow lesions where distal penetration and adaptability of the embolic agent are essential [3,4,13]. Although fragmentation could theoretically increase the risk of distal embolism, no clinical or subclinical evidence of pulmonary or arterial embolization was observed in this case series. This diffusion behavior may be beneficial in lesions where reflux is contraindicated due to the risk of ischemic complications.

Delayed agent dislodgement may also be a potential concern [2]. However, NHCA continued to advance minutes or even hours after injection into the AVM’s nidus, noting that the venous component could stretch, fragment, and migrate with no evidence of thrombosis or clinical complications. When venous passage of NHCA is observed in initial embolization cases, this property may be advantageous in staged treatment, as remnant flow from other primary veins can facilitate washout of the embolic agent from the main vein, thereby reducing the risk of postoperative bleeding related to early occlusion of the main vein. While NHCA migration may offer advantages in staged treatments, its long-term stability remains uncertain, particularly in pediatric AVMs and dural AVFs, where venous recanalization could lead to recurrence. Although no such events were observed during short-term follow-up in this case series, higher-volume studies with extended observation are needed to evaluate its long-term efficacy and safety.

Our center does not function as a pediatric referral facility; therefore, our experience with pediatric vascular malformations is limited. Nevertheless, based on available literature and the known pharmacological profile of N-hexyl cyanoacrylate (Magic Glue), there are no established contraindications for its use in pediatric populations. Consequently, we believe that NHCA may have a role within the therapeutic arsenal for selected pediatric cases, particularly when the properties of prolonged injection time and low adhesive strength are desirable.

NHCA's lower adhesive strength compared to other LEAs may help mitigate the risk of catheter entrapment compared to other cyanoacrylate embolic agents [4,5,14]. Its ability to support prolonged injections without catheter entrapment when managing complex, high-flow vascular lesions where extended delivery is critical can offer procedural advantages over traditional cyanoacrylates such as NBCA, which has high adhesive strength and rapid polymerization, particularly during complex neurovascular procedures [3,4,15]. In all procedures in this series, the catheter was successfully withdrawn without signs of entrapment or procedure-related complications. This consistent retrievability, even following prolonged injections and partial embedding of the catheter tip, allowed for the use of standard microcatheters rather than costlier, less navigable tip-detachable alternatives. 

The main limitation of this study is the heterogeneity of the included pathologies and vascular territories. While the injection technique remained consistent with other cyanoacrylates, the behavior of NHCA can only be described qualitatively based on observed outcomes. Additionally, this was a single-center descriptive case series with a relatively small sample size and no control group, limiting the strength of comparative inferences. Selection bias may also be present, as the decision to use NHCA over other embolic agents was based on operator judgment and case-specific technical considerations. Furthermore, no formal long-term follow-up was conducted, and the study did not include a direct comparison with other embolic agents such as NBCA. Despite these limitations, the descriptive observations presented here offer a preliminary foundation for future studies with more defined objectives and comparative methodologies.

## Conclusions

In our study, NHCA achieved successful embolization in 21 of 24 cases, with no clinically significant complications. Although the agent's long-term stability requires further evaluation, short-term outcomes suggest that NHCA offers an effective and safe alternative to other LEAs, particularly when prolonged injection or distal penetration is needed.
